# Early Pregnancy: And Infantile Menstruation

**Published:** 1849-08

**Authors:** 


EARLY PREGNANCY: AND INFANTILE MENSTRUATION.

In the London Medical Gazette, for 3d Nov. 1848, Mr. John Smith publishes a recent case of *Early Pregnancy.* It is interesting, not only from the extreme youth of the mother, but from the fact of her having borne a living and tolerably healthy infant. The following is Mr. Smith's narrative:—

"At the Coventry Assizes, of August, 1848, Julia Amelia Sprayson preferred a charge of rape against her uncle, James Chattaway, who was convicted of the assault, and sentenced to two years' imprisonment and hard labor in the House of Correction. The girl was far advanced in a state of pregnancy, and as it is of rare occurrence for conception to take place at so early an age as *between eleven and twelve years,* many surmises were expressed by the gossips as to what would be the probable issue. She continued in good health up to the day of delivery, which took place on the 16th September, 1848. In the early part of the morning she became restless and uneasy; and from the hour of 11, A. M., slight pains occurred at irregular intervals, until about 5, P. M., when it was evident that labor was rapidly advancing. On being sent for soon after, in consequence of the absence from town of Dr. Dewes, who had been engaged to attend her, I proceeded to make an examination, when I found the pelvis of average dimensions, and the os uteri about the size of a shilling piece; but as the parturient throes were active, and returned every eight or ten minutes, it appeared prudent to remain until the case had terminated. Nothing remarkable supervened during the progress of the labor, except that it was of unusually short duration. From first to last she was not more than ten hours ailing, while the period of actual labor was not extended beyond four hours, and this would have been further shortened but for the smallness of the external outlet. The subsequent symptoms were just as favorable as the labor had been short. The lochia ceased after the lapse of a few days: the mammæ became duly developed, and the secretion of milk was so copious as presently to suggest to her mother the idea of seeking for a situation as wet nurse. The infant at birth was long, slender, and emaciated, but rather below the average size, and in many respects may be said to have borne a striking resemblance to the offspring of mothers who had been imperfectly nourished during pregnancy. It did not occur to me at the time, either to place it in the scales, or to take its admeasurement, but at the time of writing this report (23d October, 1848,) it is 8¼ pounds in weight. The present weight of the mother is 104½ pounds. When she had so far recovered as to take a share in domestic avocations, it seemed advisable to pay her an early visit, to elicit, if possible, some farther information than what had transpired in court, with a view of establishing some data as to the period of utero-gestation; and although foiled and disappointed with the result of this part of the investigation, some particulars of interest were readily obtained. She was rather of prepossessing appearance, of fair complexion, with brown hair and dark gray eyes; more womanly by far than is usually witnessed at her age, her figure being tolerably plump, well set and proportioned, and her height being rather more than five feet; and notwithstanding her casually childish manner, there was that forwardness of expression which betokened a more than ordinary development of character. On inquiry her mother assured me that she began to menstruate when *ten years and six weeks* old; and it was distinctly ascertained that there had been a regular return of the catamenial discharge, in somewhat profuse quantity, up to the period at which conception took place. The girl had lost her father about two years ago, and that she might not be a burden to her widowed mother, had been in residence with her uncle, who was a weaver at Foleshill. This unhappy man, who proved her seducer, was aged forty-seven, living with his wife, to whom he had been married twenty-five years, and by whom he had had a family of two or three children. The niece was taught to weave at a handloom, which stood in the same apartment in which her uncle pursued his daily employment; and here it would seem that familiarities arose which issued at length in criminal intercourse. This latter took place for the first time about the middle of November, 1847, and was allowed to be repeated on four occasions at weekly intervals; but as the catamenia had appeared during the last week of that month, and did not recur in the Christmas week, she dated conception from the latter period. No communication was made to her relations of what had transpired until six months had elapsed, when her situation became too prominent to elude further observation, and then it was that arrangements were made for bringing her under the maternal roof; and means were taken for delivering her seducer into the hands of justice. The most rigid inquiry failed in deducing any farther particulars that could be at all relied on as authentic information. . . . . . I have been at the pains of consulting the registers both of her birth and baptism. The former bears the date of February 13th, 1836, and the latter March 7th, of the same year."

*Early Pregnancy.*—In connexion with the above, the following notes of cases of early pregnancy may be interesting to many; the more especially at present, when we may expect to hear of similar, or more remarkable cases, occurring in those continental cities which have lately been the scene of revolutionary license. That the aptitude of the human female for conception at a tender age is greater than is commonly imagined, we may infer from the fact that during national convulsions (in which the bonds of social order and decency have been broken,) cases of early pregnancy have been observed to be of more frequent occurrence. During the revolution in France, at the close of the last century, several instances occurred of females of eleven, and even below that age, being received, in a pregnant state, into the Maternité at Paris.

1. Sir Everard Home says, "I have met with corpora lutea in virgins at fourteen, and know of two instances of girls still earlier, one at thirteen, the other at twelve."—*Phil. Trans.* 1819. p. 61.

2. Dr. W. F. Montgomery says, that "the earliest instance of pregnancy known to him, was that of a young lady who brought forth twins before she had completed her fifteenth year."—*Signs and Symptoms of Pregnancy*, p. 163.

3. Mr. Robertson, of Manchester, mentions a case which occurred in the practice of Mr. R. Thorpe. It is thus quoted from the Edinburgh Medical and Surgical Journal, vol. xxxviii, p. 231, by Dr. Montgomery:—"She had been employed in a cotton factory, and was represented to have become pregnant in her eleventh year. Mr. Thorpe and the late Dr. Hardie were at the trouble of examining the registers of her birth and christening, and fully satisfied themselves that she had really conceived during the eleventh year of her age, and that at the time of her delivery she was only a few months advanced in her twelfth year; her figure was that of a well-grown young woman, with fully developed mammæ, and it was ascertained that she had menstruated before she became pregnant."—*Op. cit.* p. 162.

4. Dr. Rowlett, of Waisborough, Kentucky, reports, in the Transylvania Medical Journal, vol. vii., p. 447, the case of Sally Deweese, born 7th April, 1824, in the county of Butler, Kentucky. "She began to menstruate at a year old, and the pelvis and breasts became developed in an extraordinary degree: she continued to menstruate regularly up to 1833, when she became pregnant, and on the 20th April, 1834, she was delivered of a female child, weighing seven pounds and three-quarters. At the time of publishing the case the child weighed eight pounds and three-quarters, and the mother 100 pounds, and was four feet seven inches in height."—(As quoted by Montgomery, *Op. cit.* p. 162.)

5. La Motte delivered a girl who had not completed her thirteenth year, and who had never menstruated.—(*Traité des Accouchemens,* Obs. xxiii., p. 52, as quoted by Montgomery, *Op. cit.* p. 163.)

6. Dr. Michael Ryan knew of a female pregnant at 12½ years of age.—*Medical Jurisprudence,* p. 242.

*Infantile Menstruation.*—The following are a few curious instances, some of which certainly may be considered as puberty at an infantile age:—

1. Mr. Embling, in the Lancet for January 29, 1848, gives the following case:—At the date when the account was published, the child was three years old, and had during some preceding months menstruated regularly. The mammæ and nates were as fully developed as in an adult of twenty; the labia, etc. were like those of a mature young woman; the hymen was perfect; the vagina anteriorly was of large size; and on the pubes there was a slight growth of hair. The countenance, appearance and gait were in miniature those of an old woman. At her menstrual periods, she suffered the uterine, lumbar, and other pains common in women capable of utero-gestation.

2. Dr. Dieffenbach, of Berlin, in Meckel's Archiv für Anatomie, etc., 1827, p. 367, relates a case of early menstruation in a child nineteen months old. It was at birth of the natural size, but after the first month began to grow rapidly. In her ninth month she was as large as a child a year and a half old; and about this time a discharge of blood from the vagina was observed. At the end of two months a more copious discharge took place, which was accompanied with an increase in the size of the mammæ, and the appearance of hairs on the genitals. The same phenomenon recurred at fourteen, and again at eighteen months. At the time of the report the mammæ were large, and the genitals were largely developed and covered with hairs. Nothing was remarked in her mental disposition different from other children of the same age, and there was no indication of sexual desire.

3. Dr. Catals, of Adge, attended a little girl of six years old, who was affected with a spasmodic cough, colic, headache, and epistaxis, which recurred every month. With other remedies which this condition indicated, he applied leeches to the calves of the legs. A discharge of blood from the uterus supervened, which was preceded by a febrile state. These phenomena, accompanied with some enlargement of the mammæ, pain in the lumbar region, and itching of the genitals, returned regularly every month, and lasted three days.—(*Journal de Médecine et de Chirurgie*, par Corvisart, Leroux, et Boyer, t. xi. p. 37, as quoted by De Boismont, in his work, *De la Menstruation*, p. 33. Paris: 1842.)

4. M. A. Brierre De Boismont, op. cit. p. 35, relates, on the authority of M. Le Beau, the case of Matilda H., who was born at New Orleans in 1827, with the breasts and genitals as perfectly developed as in a girl of 13 or 14 years. The menses appeared regularly each month, from the age of three years. They continued three days; and were as copious as in a perfect woman. At the age of four years, when the report was made, she was well-formed, and of handsome appearance; the mammæ were of the size of a large orange; and the pelvis seemed as large as in a child of eight years. Her health was excellent.—(From *Annal. d'Hygiène*, t. x. p. 484.)

5. Dr. Carus, of Dresden, mentions the case of Christina Theresa, born in the mountains of Saxony, of parents of a weak constitution. She was scarcely a year old when she began to grow rapidly. At the end of the second twelve-month the catamenia appeared, and continued to flow regularly once a month. The mammæ were firm, like those of a strong girl of 16; the body was stoutly made; and the genital organs were covered with dark brown hair. Her intellectual functions, tone of voice, and physiognomy, were those of a child three years old.—(*Allgemeine Zeitung für Chirurgie*, as quoted in *Edinburgh Monthly Journal of Medical Science*, p. 1050. 1842.)

6. Mr. W. H. Whitmore, of Cheltenham, communicated to the Northern Journal of Medicine, for July, 1845, an account of the case of a child who menstruated regularly, at intervals of three weeks and two or three days, from a few days after birth, until the age of four years and some months, when she died. The development of the body equalled that of a girl 10 or 11 years of age. The mammæ were unusually large; the mons veneris well covered with hair; the labia pudendi more sparingly so. In the absence of her periodical ailments, she would enter into the amusements of children of her own age; but when she was indisposed, she was exceedingly reserved, and would withdraw from all her playful occupations.

7. Dr. Lenz, of Dantzig, relates a case in which menstruation appeared at the eighteenth month, and continued up to the age of two years, when the case was reported. The general health was unaffected in the intervals, provided the discharge took place at the regular periods. The breasts and genital organs presented no remarkable appearance, but experienced an increase in temperature and size at each menstruation.—(*Caspar's Wochenschrift*, Oct. 3, 1840.)

8. M. Gruere, of Dijon, was acquainted with the case of a child, aged three years, who had menstruated regularly since she was one year old. Her general health was good. There were no premonitory symptoms, except a slight feeling of tension in the hypogastric region. There were no external signs of puberty.—(*Journal de Medecine et de Chirurgie Pratique.* Mai, 1842. Paris.)

In addition to the above cases, others have been recorded, in which a discharge of blood, often accompanied with some enlargement of the breasts, took place from the genital organs soon after birth. It seems probable, however, that the hæmorrhage might have arisen from other causes than the establishment of menstruation; and that the enlargement of the mammæ may be due to the sympathy which exists between them and the genital organs, independent of sexual aptitude. Of this kind are, probably, among others, the cases recorded by M. Mallat in the Gazette Médicale for 1832; and by Dr. Camerer in the Medicinisches Correspondenz-Blatt, as quoted in Gazette Médicale, p. 248. 1815.—*Ibid.*

